# Factors Influencing the Clinical Course of Bullous Pemphigoid among Geriatric Patients: A Pilot Study

**DOI:** 10.3390/medicina60101701

**Published:** 2024-10-16

**Authors:** Paula Mazan, Aleksandra Lesiak, Igor Bednarski, Dorota Sobolewska-Sztychny, Marcin Noweta, Joanna Narbutt

**Affiliations:** 1 Department of Dermatology, Pediatric Dermatology and Oncology, Medical University of Lodz, 90-419 Lodz, Poland; aleksandra.lesiak@umed.lodz.pl (A.L.);; 2 Department of Neurology and Stroke, Medical University of Lodz, 90-419 Lodz, Poland

**Keywords:** bullous pemphigoid, elderly, pruritus, neurological diseases, comorbidities

## Abstract

*Background and Objectives*: Bullous pemphigoid (BP) is the most common autoimmune blistering disease affecting mainly elderly patients. Still, little is known about the pathogenesis of pruritus in BP or the factors that affect the clinical course of the disease. This study aimed to evaluate the factors influencing the clinical course of BP among older patients. *Materials and Methods*: A retrospective analysis of medical records of 55 patients with BP hospitalized in the dermatology department in 2015–2021 was conducted. The study focused on preliminary diagnosis, medical history, clinical examination (characteristics and location of cutaneous changes), laboratory investigation, and direct and indirect immunofluorescence. *Results*: Analysis of laboratory results in combination with the clinical course of BP showed that red blood cell count, hemoglobin, and hematocrit values were negatively associated with a risk of erosions and erythema, while MCHC values were positively correlated with a risk of associated pruritus. A correlation was found between neurological diseases and an increased risk of erosions. *Conclusions*: We have shown that age and neurological conditions, including stroke, affect the clinical course of BP. Further studies on a larger group of patients should be conducted to investigate the different factors affecting the clinical aspect of BP and to understand the relationship between them.

## 1. Introduction

**Figure 1:**
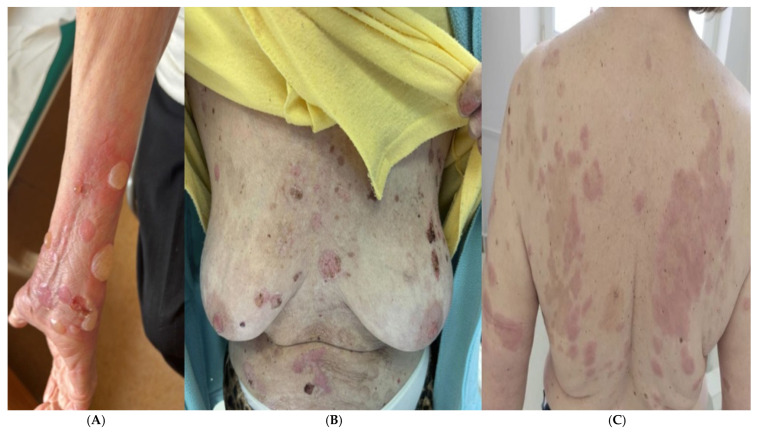
(**A**) Erythematous lesions, tense bullae, and erosions on the upper right limb. (**B**) Erythematous lesions and erosions on the chest and abdomen. (**C**) Erythematous and oedematous lesions on the limbs and back. Photographs sourced from Department of Dermatology, Pediatric Dermatology and Oncology, Medical University of Lodz, Lodz, Poland.

Bullous pemphigoid (BP) is an autoimmune subepidermal blistering disease that mainly affects elderly patients [1]. Pemphigoid diseases include mucous membrane pemphigoid (MMP), pemphigoid gestationis (PG), lichen planus pemphigoides (LPP), and anti-p200 pemphigoid. Although there have been case reports of BP occurring in children and adolescents [2,3], it is more commonly found in older individuals. The disease typically presents as a generalized pruritic bullous skin eruption, with polymorphic lesions being a possibility. However, its typical and most common form involves the eruption of inflammatory skin lesions. These lesions progress from erythema through to urticarial plaques, blisters, and erosions and then to uninflamed, scarless re-epithelizing skin (Figure 1A–C). These blisters are tense, may be up to several centimeters in diameter, and can appear either on an erythematous background or on normal skin of the limbs and trunk [2]. During the early prodromal phase of BP, the lesions may be non-specific, and bullae or vesicles may be absent. Patients may present with a range of symptoms, including mild to severe pruritus, in isolation or in conjunction with excoriated, eczematous, papular, or urticarial eruptions that may persist for several weeks or even months [4]. When blisters are absent, a diagnosis of the atypical variant of the disease, non-bullous pemphigoid (NBP), is made. NBP is characterized by pruritus and a range of skin manifestations such as urticarial plaques and papules or nodules, which can resemble other pruritic skin disorders [5,6]. Blisters are only present in 10% of cases [7], which can delay diagnosis [8].

BP is immunologically characterized by the presence of autoantibodies that recognize the BP180 (180 kDa, also called COL17) and BP230 (230 kDa) self-antigens in the basement membrane zone (BMZ). These proteins are hemidesmosome components responsible for dermal–epidermal cohesion [2]. Patients with BP have serum-circulating immunoglobulin (Ig) G autoantibodies that bind to NC16A—the main antigenic epitope in BP, which is also an extracellular domain of BP180—and to the globular C-terminal domains of BP230. Autoantibodies bind to the epitopes, causing complement activation and neutrophilic chemotaxis. This leads to the release of proteases and elastases that disrupt the BMZ and result in blister formation [9].

**Figure 2:**
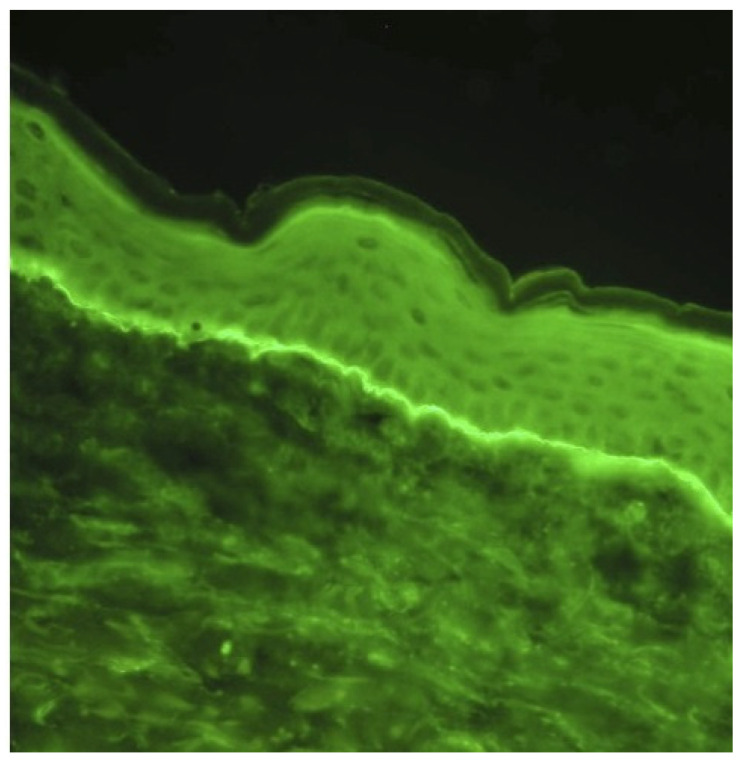
DIF demonstrated linear depositions of IgG (++) and C3 (+++) complement along the dermo–epidermal junction. Photograph courtesy of Department of Dermatology and Venereology, “Military Medical Academy” University Teaching Hospital, Lodz, Poland and Department of Dermatology, Pediatric Dermatology and Oncology, Medical University of Lodz, Lodz, Poland.

**Figure 3:**
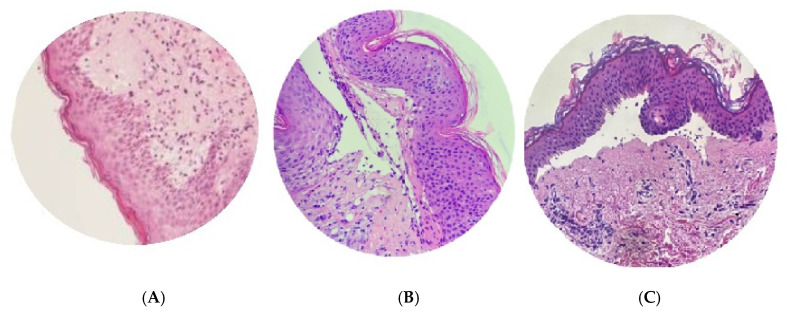
Histopathologic findings of BP in three different patients. (**A**) Epidermal spongiosis and increased dermal eosinophils (hematoxylin and eosin [H&E], ×400). (**B**) Subepidermal blister, filled with fluid and inflammatory cells, without features of acantholysis (hematoxylin and eosin [H&E], ×400). (**C**) Dermal–epidermal split with eosinophils within the blister fluid ([H&E], ×100). Photographs sourced from Department of Dermatology, Pediatric Dermatology and Oncology, Medical University of Lodz, Lodz, Poland.

BP is diagnosed based on clinical presentation, linear deposits of IgG and/or complement C3 identified at the dermal–epidermal junction using direct immunofluorescence (DIF) microscopy on perilesional skin biopsy (Figure 2) and the detection of serum autoantibodies to BP180 and/or BP230. Subepidermal blisters appear in microscopic images in these cases, accompanied by an inflammatory infiltrate composed mainly of eosinophils (Figure 3).

There are several known risk factors for BP, the most important of which is age. The mean age at BP presentation ranges from 66 to 83 years [10]. Neurological diseases, such as dementia, Parkinson’s disease, stroke, and cerebrovascular disease [11], and certain systemic medications including loop diuretics, spironolactone, dipeptidyl peptidase-IV inhibitors, and neuroleptics, are also associated with BP [12]. Dermatoses such as psoriasis or lichen planus and BP have also been found to coexist [13,14]. The incidence of BP is increasing due to factors such as increasing life expectancy, the use of certain medications, heightened awareness of BP (especially its atypical variants), and improved diagnostic methods. This disease is potentially fatal, with one-year and three-year mortality rates estimated to be 22.4% and 39.5%, respectively, in Polish patients [15]. Several prognostic factors that may increase mortality in BP patients have been identified, including advanced age, the co-occurrence of neurological disease (especially dementia), treatment with oral corticosteroids or high dosages of them, and a poor general condition/being bedridden. Other mortality risk factors for BP include female gender, disease severity, delayed diagnosis, coronary artery disease, heart failure, malignancy, diabetes, and the use of more than six medications [15].

On this basis, our study aimed to evaluate the factors influencing the clinical course of BP among geriatric patients.

## 2. Materials and Methods

In this retrospective study, the case records of 55 patients (22 males and 33 females) were analyzed, focusing on their preliminary diagnosis, medical history, clinical examination (the type and location of skin lesions), and laboratory investigations, which included the use of direct and indirect immunofluorescence. Patients over the age of 65 with a diagnosis of BP were included. The diagnosis of BP was based on a clinical examination and positive direct immunofluorescence results (DIF) in the perilesional skin indicating the presence of linear IgG and/or C3 deposits along the BMZ. Indirect immunofluorescence (IIF) was performed to detect circulating anti-BMZ antibodies, as well as to examine salt-split skin. This study was approved by the local Bioethics Committee of the Medical University (RNN/263/21/KE 14 December 2021).

Statistical analysis was performed using GraphPad Prism 9.1 and Statistica 13 software. Continuous variables are presented using the mean and standard deviation, while numerical and non-continuous variables are shown as the number of cases (N) and percentages. The distribution of variables was evaluated using the Shapiro–Wilk test. To compare differences between groups, Welch’s and Mann–Whitney U tests were used for continuous variables, while χ^2^ tests (Pearson’s and Yates’ tests) were employed for categorical variables. A univariate logistic regression model was used to identify which clinical parameters could influence the clinical picture of the disease. *p* < 0.05 was deemed significant for all analyses.

## 3. Results

### 3.1. Patient Characteristics and Laboratory Results

This study included patients with a mean age of 78.95 ± 8.18 years (range: 65–96 years) with no significant difference between genders (men: 76.68 ± 8.52 years; women: 80.45 ± 7.70 years). Patients under the age of 65 (*n* = 11; age range: 52–64 years) were excluded from this study. A total of 55 patients were included, with 22 in the age range of 65 to 75 years old, 21 in the age range of 76 to 85 years old, and 12 above the age of 85. The mean lesion duration prior to hospitalization was 3.09 months, with no significant difference between genders (Table 1). Skin lesions occurred less than a month before hospitalization in 31.81% of patients, and 16.66% of them had received a previous diagnosis of BP.

In the laboratory tests, the 55 patients were found to have slightly lower red blood cell counts and higher blood glucose and creatinine values. The men had significantly higher blood glucose, AST, ALT, hemoglobin, hematocrit, MCV, and MCH values than the women (*p* < 0.05) (see Table 1). In 16% of our patients, we observed elevated tumor markers (CEA, AFP, Ca 19–9, or PSA) or abnormalities in the imaging studies, particularly in chest X-ray examinations.

### 3.2. Diagnosis

The diagnosis of BP was confirmed using immunological tests. All patients had positive direct immunofluorescence (DIF) results for linear staining of IgG and/or C3 along the BMZ. In 59.09% of cases, the DIF results were positive for linear staining of both IgG and C3 along the BMZ, while linear staining of IgG or C3 alone along the BMZ was detectable in 27.72% and 18.18% of cases, respectively. In this study, indirect immunofluorescence (IIF) was also used to evaluate the presence of circulating IgG anti-BMZ autoantibodies. These results showed that 87.88% of the women and 88.89% of the men had detectable IgG anti-BMZ autoantibodies, with titers ranging from 10 to 640.

### 3.3. Lesion Morphology

There was no significant difference in the morphology of skin lesions between men and women. Erythematous lesions were most frequently observed in women (93.75%), while blisters were commonly observed in men (86.36%). Although pruritus occurred more frequently in women (73.33%) than men (55.56%), the gender differences were not statistically significant. Detailed data are presented in Table 2. The pruritus NRS was used to assess the intensity of pruritus in most of the patients. Based on the criteria from the literature, the intensity of pruritus was assessed as moderate (with values between 4 and 6 points). In four patients (6%), the main symptoms were pruritic skin and/or erythematous or erosive lesions, while blisters were absent.

Studying the relationship between age and the clinical course of BP revealed that there was a much higher incidence of erosions in patients over the age of 85, while patients aged 65–75 and 76–85 were more likely to have blisters. Table 3 presents the detailed data.

**Figure 4:**
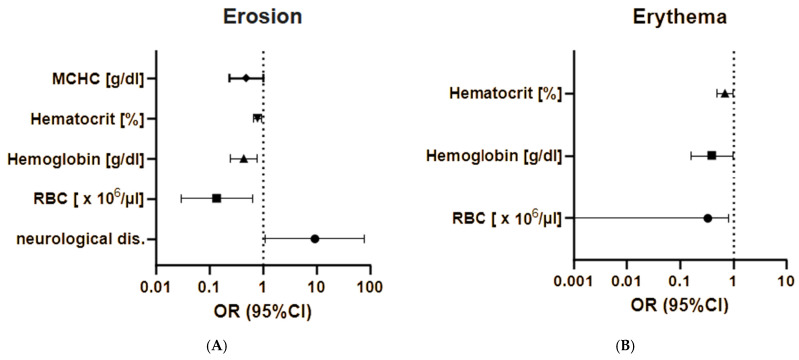
Correlations between parameters of the red blood cell system and risk of erosions (**A**) and erythema (**B**). RBC—red blood count, MCHC—mean corpuscular hemoglobin concentration.

**Figure 5:**
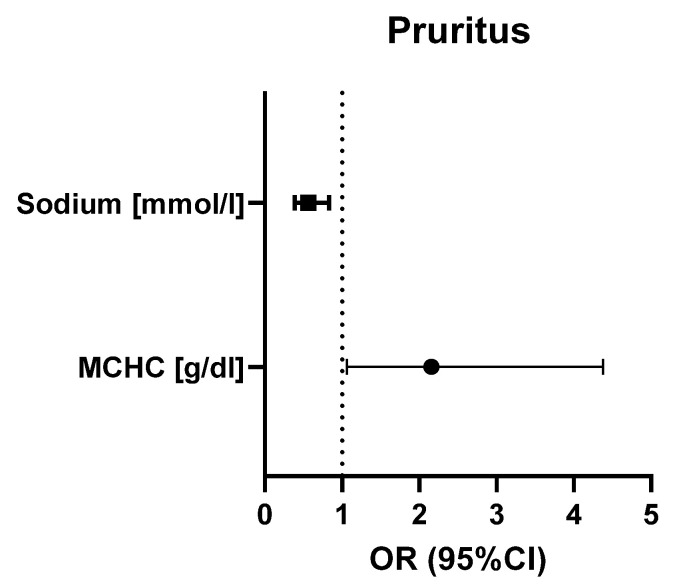
Correlation between MCHC, sodium, and pruritus. MCHC—mean corpuscular hemoglobin concentration.

Univariate logistic regression revealed several correlations in our patients. Higher red blood cell system parameters, including the red blood cell count (OR = 0.1, 95% CI = 0.00–0.6, *p* = 0.0114) and hemoglobin (OR = 0.4, 95% CI = 0.2–0.8, *p* = 0.0006), hematocrit (OR = 0.8, 95% CI = 0.7–0.9, *p* = 0.0113), and MCHC values (OR = 0.5, 95% CI = 0.2–1.0, *p* = 0.0497) were associated with a significantly reduced risk of erosions (see Figure 4A). Equally, the risk of erythema was significantly reduced with a higher red blood cell count (OR = 0.0, 95% CI = 0.00–0.8, *p* = 0.0362) and higher hemoglobin (OR = 0.4, 95% CI = 0.2–1.0, *p* = 0.0440) and hematocrit values (OR = 0.7, 95% CI = 0.5–1.0, *p* = 0.0434) (Figure 4B). Meanwhile, higher MCHC values were associated with a higher risk of accompanying pruritus (OR = 2.2, 95% CI = 1.1–4.4, *p* = 0.0332), while higher sodium levels were significantly correlated with a reduced risk of this symptom (OR = 0.6, 95% CI = 0.4–0.8, *p* = 0.0036) (Figure 5).

### 3.4. Comorbidities

**Figure 6:**
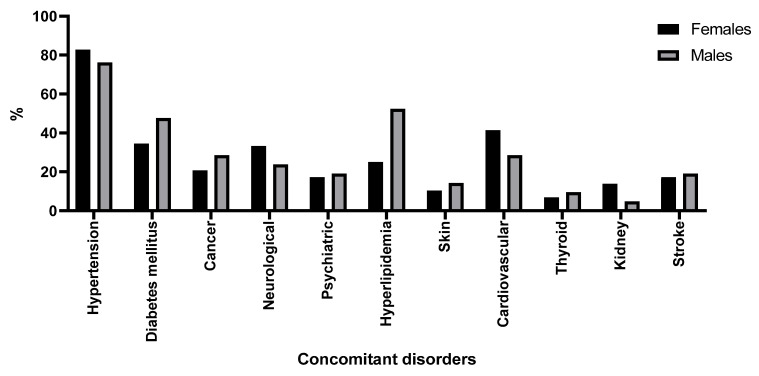
Review of comorbidities in female and male patients with BP.

This study identified hypertension as the most prevalent comorbidity in our cohort, affecting 82.76% of the women and 76.19% of the men. The second most common comorbidity in women was cardiovascular disease other than hypertension, while hyperlipidemia was more prevalent in men. Neurological diseases (including Parkinson’s disease, dementia, stroke, multiple sclerosis, epilepsy, and polyneuropathies) were observed in 33.33% of the women and 23.81% of the men, with stroke reported in 17.24% of women and 19.05% of men. Additionally, neurological diseases were associated with an increased risk of erosions (OR = 9.3, 95% CI = 1.1–79.2, *p* = 0.0406) as shown in Figure 4A. BP-associated dermatoses were observed in 10.34% of the women and 14.29% of the men (Figure 6).

### 3.5. Treatment

No statistically significant differences were observed in the treatment applied between female and male patients. Systemic steroid therapy was the most common treatment for women, followed by methotrexate. For men, topical steroid preparations were the most common, followed by systemic corticosteroids and methotrexate. Methotrexate was administered at doses of 7.5 mg to 15 mg per week. Tetracycline was used in combination with vitamin PP in 24.24% of the women and 27.27% of the men. Only a small number of patients received antibiotics other than tetracycline (Table 4).

While topical steroids were significantly more frequently used in patients over the age of 85, no statistically significant differences were found in the use of the other treatments. Topical and systemic steroids were the most common treatment for patients aged 65–75 years old, while systemic steroids were the preferred treatment for those aged 76–85 years old. Methotrexate was frequently administered to patients aged 65–75 (Table 5).

## 4. Discussion

In our cohort of patients diagnosed with BP, hospitalization was more common among women, with an average age of 79 years old, a finding consistent with the previous literature. BP typically affects patients over 75 years old, with some researchers suggesting a higher prevalence in women [16]. However, Miyamoto et al. [1] reported no gender-related difference, and some studies indicated that once they are over the age of 80, men are more likely to suffer from BP than women [17]. In the group of patients analyzed, 21 individuals (33.33%) were over 80, 15 (71.42%) of whom were female. The majority of the data suggest no gender-related discrepancy in BP prevalence.

Three or more body parts were affected by skin lesions in just over half of the patients examined, indicating a disseminated form of BP. The skin lesions were mainly tense bullae and erythematous lesions. Pruritus was reported in 73.33% of women and 55.56% of men. As the literature describes, this disease can manifest in two forms, localized or disseminated [1], and it often affects the flexural aspects of the limbs and the abdomen [2]. Pruritus is present in nearly all patients with typical skin symptoms and may also be the primary symptom during the prodromal phase of the disease, preceding typical lesions by several weeks or months [2]. The discrepancy between the prevalence of pruritus observed in the patient group studied and that suggested by the data from the literature may be attributed to inaccuracies in the patients’ medical histories or the omission of this symptom from their medical records. Moreover, the medical records did not include information on pruritus as a symptom preceding characteristic skin lesions. However, in four patients (6%), blisters were not observed, and the main symptoms were pruritic skin and/or erythematous or erosive lesions. These findings are consistent with those reported in the literature [18].

Our laboratory test analysis revealed statistically significant differences between the women and men examined, with higher hemoglobin, hematocrit, MCV, MCH, glycemia, and transaminase values observed in the men. This variation in red blood cell parameters is due to the difference in the mean hemoglobin levels between men and women. Women have mean levels approximately 12% lower than men, which is likely a direct effect of sex hormones on erythropoiesis and independent of iron status [19]. Furthermore, women tend to have lower serum aminotransferase concentrations than men [20]. Few studies in the literature have analyzed the laboratory parameters in patients with BP, making it challenging to identify similar observations in other studies conducted to date. The literature suggests that BP patients may have elevated serum total IgE levels and/or be diagnosed with peripheral eosinophilia only [21]. As previously mentioned, analyzing the laboratory results and the clinical course of BP in our patients revealed several correlations, including a negative association between the risk of erosions and erythema and the red blood cell count, hemoglobin, and hematocrit values. The lower risk of erosions and erythematous lesions in patients with higher red blood cell system parameters may be due to better healing and overall better health. However, in recent reports on the establishment of physiological norms for the red cell system in the elderly population, Zierk et al. found that hemoglobin and red blood cell counts decrease with age in men, while this trend is only observed in women after the age of 80 [22]. The study’s authors argue that lower reference ranges for red blood cells in older people are a physiological phenomenon. The patients’ results were interpreted in accordance with the established sex-dependent norms for adults, and it should be noted that slightly lower red blood cell counts were observed. Remarkably, analysis of the characteristics of pemphigoid symptoms in relation to the age of our patients showed that erosions were statistically significantly more frequent in patients over the age of 85 (and therefore in patients with lower hemoglobin and RBC values) than in younger age groups. These observations, combined with the observations shown and described above regarding the significantly statistically higher risk of developing erosions in patients with lower red blood cell system parameters, appear to be consistent. Thus, analysis of the results according to age subgroups reveals that these red blood cell abnormalities, typical of the geriatric population, may potentially contribute to the development of pemphigoid, which undoubtedly may be an important observation from the point of view of the pathogenesis of pemphigoid. Although there was no significant difference in the morphology of skin lesions between men and women in our study, erosions were observed significantly more often in women (who described statistically significantly lower hemoglobin and MCH values) than in men (*p* = 0.0567), which also seems to confirm the influence of red blood cell system parameters on the development of pemphigoid symptoms.

The research community agrees that the pathogenesis of pruritus in BP is complex and requires further investigation. It has been demonstrated that for patients with BP, pruritus harms their quality of life [23]. Consequently, targeted treatment is essential to address this significant issue [24]. The results of our study indicated a positive correlation between mean corpuscular hemoglobin concentration (MCHC) levels and the incidence of pruritus, while serum sodium levels were negatively correlated with pruritus risk. Dehydrated patients frequently exhibit elevated MCHC values, and dry skin can exacerbate pruritic complaints [25]. One study on chronic kidney disease-associated pruritus found that patients with pruritus exhibited lower serum sodium levels than those without pruritus [26]. Based on the available literature, eosinophils, IgG autoantibodies, IgE autoantibodies, SP and its receptor NK1R, IL-31 and its receptor OSMRb, IL-31RA, IL-13, IL-4, periostin, and basophils may be responsible for pruritus in BP and could be potential therapeutic targets [27]. While a number of studies have indicated that mast cells may play a role in BP pathogenesis and BP-associated itching [28,29], recent studies have challenged this hypothesis, suggesting that mast cells do not play a role in pruritus induction in BP patients [30]. Efforts are being made to establish a relationship between hematological inflammatory biomarkers, such as the neutrophil-to-lymphocyte ratio and the platelet-to-lymphocyte ratio, and disease activity in BP. However, the current findings are unclear, requiring further analysis. Hematological inflammatory biomarkers are recognized markers of systemic inflammation and have prognostic value in various other diseases, including systemic lupus erythematosus, rheumatoid arthritis, cutaneous polyarteritis nodosa, Behçet’s disease, IgA vasculitis, pemphigus vulgaris, and psoriasis [31].

Extensive research has been conducted on the relationship between BP and other diseases, including diabetes mellitus, neoplasms, and neurological, cardiovascular, dermatological, and other autoimmune diseases. In our study group, arterial hypertension was the most prevalent comorbidity identified, which is consistent with the findings of several previous studies, including Kremer et al., Kwan et al., and Kalinska-Bienias et al., which found arterial hypertension to be the most frequent comorbidity in BP patients (at 64%, 62.8%, and 76.1%, respectively) [32,33,34]. The patients in our study frequently exhibited cardiovascular disease, diabetes, and hyperlipidemia as well. The relationship between BP and cardiovascular diseases (CVDs) [35,36] or diabetes mellitus (DM) is well established in the literature [37,38]. Diabetes mellitus (DM) and cardiovascular diseases (CVDs) are the most frequent comorbidities and are associated with higher mortality rates [39]. Furthermore, 18.18% of the women and 21.05% of the men in our study were diagnosed with malignant neoplasms prior to the onset of BP skin lesions. On the other hand, the association between BP and malignancies is considered controversial as the incidence of cancer in patients with BP has not been definitively established [40]. A meta-analysis by Lucariello et al. [41] revealed an 11% malignancy rate among BP patients in 16 studies.

This study revealed that BP is linked to specific dermatological conditions, including psoriasis and lichen planus, with blisters that may be located within psoriatic plaques or lichenoid papules. In our study, BP-associated dermatoses were observed in 12.12% of the women (with two cases of coexisting psoriasis, one case of coexisting Grover’s disease, and one case of coexisting venous ulcers) and 21.05% of the men (with three cases of coexisting psoriasis and one case of coexisting Grover’s disease). Several studies demonstrate a correlation between BP and a range of neurological conditions, including Parkinson’s disease, Alzheimer’s disease, multiple sclerosis, and stroke. In our study of patients with BP, we observed that 33.33% of the women and 23.81% of the men had developed neurological diseases (NDs). The most prevalent neurological condition observed in our cohort was dementia, which was diagnosed in three women and two men. Epilepsy was the second most common condition, diagnosed in two men and one woman. One woman and one man were diagnosed with Parkinson’s disease. Additionally, the female patient group included one case each of Alzheimer’s disease, multiple sclerosis, and muscle tremor. In four cases comprising three men and one woman, stroke coexisted with another neurological disease. In 17.24% of the women and 19.05% of the men, stroke preceded the development of skin lesions. These findings are consistent with previous studies that reported rates of neurological diseases in BP patients ranging from 23% to 73.8% [42,43,44]. A review of the literature indicates that the prevalence of stroke in BP is estimated to be between 7.7% and 44.4% [45,46]. In a systematic review by Milani-Nejad et al., patients with BP and concomitant diagnoses of stroke and dementia exhibited a significantly higher one-year mortality rate [11], with these authors reporting an average time between the onset of BP and the diagnosis of a neurological disease of 6.7 years [11]. Moreover, we observed a significant correlation between the presence of neurological diseases and an elevated risk of erosions in our BP patients, consistent with the pathophysiological mechanisms of BP and the existing literature [47]. In BP, the formation of inflammatory lesions is caused by autoantibodies binding to epitopes, triggering complement activation and neutrophil chemotaxis. This results in the release of proteases and elastases that subsequently destroy the BMZ. Epidemiological studies demonstrate that neurological disorders are a risk factor for BP [46,48] and that BP autoantigens have been identified in the central nervous system. Studies show that COL17 is expressed in numerous brain cells [49,50], and anti-COL17 autoantibodies were identified in 18% of patients with Alzheimer’s disease (AD) [51]. This suggests that neuroinflammation may cause an immune response in both neural and cutaneous antigens [52,53]. Furthermore, elevated anti-COL17 NC16A autoantibody levels were found to be associated with more severe dementia in AD, further substantiating the link between BP and neurodegenerative diseases. It is also pertinent to consider epitope spreading, whereby the cellular and/or humoral immune response targets may expand over time from the initial dominant epitope to other epitopes on the same protein (intramolecular epitope spreading), or to other proteins in the same tissue (intermolecular epitope spreading). Epitope spreading has been described in both bullous pemphigoid and neurological diseases, including multiple sclerosis and myasthenia gravis [54]. A multicenter study prospectively demonstrated that 17 out of 35 (49%) patients with BP exhibited epitope spreading, which occurred predominantly in the early stages and was linked to disease severity [55]. Due to the retrospective nature of our study and the lack of follow-up, it is challenging to assess the mortality risk in patients with BP and neurological diseases or any other comorbidities.

Four patients were diagnosed with drug-associated BP over the course of this study. Two patients exhibited cutaneous lesions a few days after antibiotic treatment, while one developed such lesions following the administration of torasemide. The latter patient had also taken oral antidiabetic medication shortly before the first symptoms appeared and was treated with phototherapy for psoriasis. There are existing case reports of drug-associated BP in the literature, and the list of drugs known to cause BP-like skin lesions is continually expanding, including antibiotics, diuretics, beta-blockers, neuroleptics, and non-steroidal anti-inflammatory drugs (NSAIDs) [56]. Moreover, the role of DPP4 [57] and biological drugs such as TNFalpha inhibitors is increasingly being emphasized [58]. Moreover, physical factors such as phototherapy alone or in combination with drug intake can also induce BP [59,60].

A review of the literature reveals the existence of case reports of BP induced by vaccination against a number of infectious diseases, including tetanus, diphtheria, poliomyelitis, pertussis, influenza, meningococcus, pneumococcus, hepatitis B, rabies, and BCG [61]. The relationship between SARS-CoV-2 vaccination and BP remains unclear and has been the subject of several studies, with some suggesting a potential association between SARS-CoV-2 vaccines, in particular mRNA vaccines, and the occurrence of BP [61]. Given that the Polish national SARS-CoV-2 vaccination program began on 27 December 2020, we did not incorporate SARS-CoV-2 vaccination status into our study.

A comparison of the clinical course of BP and the treatment provided in relation to the age of the patient population studied revealed two significant correlations. In the oldest subgroup of patients, those over 85, erosions were significantly more frequent, and the use of topical steroid drugs was significantly more prevalent. Notwithstanding the immunological aspects, the evolution of changes, and the repair mechanisms observed in BP, it is notable that several skin disorders develop as the skin ages. The dermis deteriorates, with a reduction in the thickness of collagen and elastin fibers and an increase in their disorder; furthermore, a gradual atrophy of blood vessels is observed. Such alterations can render elderly patients more susceptible to vascular disorders and skin injuries. Additionally, the skin’s capacity to repair itself gradually declines. Some treatment recommendations for BP appear to corroborate our observations. One study comparing the use of clobetasol cream and systemic steroids demonstrated that 0.05% clobetasol cream effectively controlled skin lesions in moderate BP, regardless of the daily dose, and reduced the number of side effects and mortality risk [1]. However, the use of topical clobetasol is not without limitations, including its potential to cause systemic side effects and exacerbate skin atrophy, particularly in older patients, due to difficulties with self-application. In some BP treatment guidelines, systemically administered medications remain the recommended first-line treatment for more severe cases of BP. In accordance with recent recommendations [62], however, topical treatment is now considered the optimal initial treatment in severe cases. High-potency topical corticosteroids have been demonstrated to achieve clinical disease activity (CDA) resolution more rapidly than oral prednisolone 1 mg/kg/day, with fewer severe side effects and lower mortality rates. Treatment plans for BP should be tailored to individual patients, taking into account their age, concomitant illnesses, general condition, and ability to care for themselves. It is crucial to acknowledge that BP primarily affects older patients, who frequently present with multiple comorbidities and polypharmacy.

## 5. Conclusions

This study showed that certain laboratory parameters, including electrolyte balance and elements of the red blood cell system, were associated with an increased risk of symptoms in the course of BP. It is possible that these parameters, which are somewhat correlates of the patient’s condition, indicate that a lack of compensation in terms of normal hemoglobin concentration and normal red blood cell count initiates the reaction leading to the development of pemphigoid. Higher MCHC and lower sodium levels are linked to increased pruritus, indicating that hydration status and red blood cell health are crucial. Aligning patients’ red blood cell parameters and sodium metabolism and closely monitoring these aspects could help reduce disease symptoms and improve lesion healing. Furthermore, age and neurological status were found to be determinants of the clinical course of BP among our older patients. Our observations regarding pruritus as the only symptom of BP and the association between BP and neurological disease and/or preceding stroke are consistent with the existing literature. Patients with neurological disease and chronic pruritus require close monitoring and a BP diagnosis to facilitate early and prompt treatment. Improving diagnostic and therapeutic processes may potentially reduce mortality in patients with BP. Our conclusions underscore the complex interplay of demographic, clinical, and laboratory factors in BP, highlighting the need for personalized patient care.

### Limitations

The retrospective nature of the study and the relatively small sample size of BP patients may have influenced the final results and conclusions.

## Figures and Tables

**Table 1:** Patients’ characteristic and laboratory results.

	Whole Group (*n* = 55)	Females (*n* = 33)	Males (*n* = 22)	*p*-Value
**Age (years)**	78.95 ± 8.18	80.45 ± 7.70	76.68 ± 8.52	0.0527
**Lesion duration (months)**	3.09 ± 5.17	3.45 ± 6.51	2.55 ± 1.96	0.3346
**WBC [×10^3^/μL]**	8.80 ± 3.38	8.76 ± 3.57	8.85 ± 3.13	0.5148
**RBC [×10^6^/μL]**	4.24 ± 0.57	4.13 ± 0.53	4.40 ± 0.61	0.0504
**Hemoglobin [g/dL]**	13.08 ± 1.82	12.54 ± 1.74	13.93 ± 1.62	0.0037
**Hematocrit [%]**	39.53 ± 5.18	38.08 ± 4.97	41.81 ± 4.76	0.0041
**MCV [fl]**	93.48 ± 4.70	92.25 ± 4.07	95.40 ± 5.07	0.0406
**MCH [pg]**	30.92 ± 1.92	30.34 ± 1.82	31.82 ± 1.76	0.0134
**MCHC [g/dL]**	33.08 ± 0.98	32.89 ± 1.05	33.37 ± 0.78	0.1191
**PLT [×10^3^/μL]**	246.54 ± 75.81	254.24 ± 78.10	234.43 ± 72.25	0.3501
**Neutrophiles [×10^3^/μL]**	5.51 ± 2.72	5.43 ± 2.89	5.64 ± 2.49	0.4383
**Lymphocytes [×10^3^/μL]**	1.74 ± 0.77	1.85 ± 0.76	1.57 ± 0.76	0.1970
**Eosinophils [×10^3^/μL]**	0.82 ± 1.09	0.82 ± 1.05	0.83 ± 1.18	0.6728
**Glucose [mg/dL]**	118.57 ± 51.86	104.85 ± 21.39	138.39 ± 73.66	0.0245
**Sodium [mmol/L]**	139.98 ± 3.79	140.55 ± 3.80	139.06 ± 3.69	0.0707
**Potassium [mmol/L]**	4.38 ± 0.52	4.32 ± 0.55	4.48 ± 0.45	0.4520
**Creatinine [mg/dL]**	1.00 ± 0.30	0.95 ± 0.28	1.08 ± 0.33	0.1468
**ALT [U/L]**	17.81 ± 16.23	13.18 ± 7.80	25.10 ± 22.57	0.0011
**AST [U/L]**	19.13 ± 11.26	16.44 ± 5.42	23.45 ± 16.15	0.0289
**TSH [mg/dL]**	1.65 ± 1.18	1.54 ± 1.19	1.79 ± 1.28	0.6282
**Cholesterol [mg/dL]**	169.79 ± 31.92	167.00 ± 37.08	174.44 ± 21.99	0.5985
**LDL [mg/dL]**	92.09 ± 30.14	89.71 ± 34.31	95.78 ± 23.67	0.9754
**HDL [mg/dL]**	56.85 ± 19.76	52.84 ± 22.26	63.10 ± 14.00	0.1794
**Triglycerides [mg/dL]**	96.25 ± 24.39	100.17 ± 24.82	90.38 ± 24.11	0.4269

WBC—white blood count, RBC—red blood count, MCV—mean corpuscular volume, MCH—mean corpuscular hemoglobin, MCHC—mean corpuscular hemoglobin concentration, PLT—platelet count, ALT—alanine aminotransferase, AST—aspartate aminotransferase, *p*-value < 0.5.

**Table 2:** Characteristics of the lesion morphology in women and men.

Lesion Morphology	Females	Males	*p*-Value
**blisters**	81.82% (27)	86.36% (19)	0.6553
**papules**	15.15% (5)	14.29% (3)	0.7599
**erosions**	81.25% (26)	57.14% (12)	0.0567
**pruritus**	73.33% (22)	55.56% (10)	0.3428
**erythema**	93.75% (30)	85.00% (17)	0.2978

**Table 3:** Lesion morphology characteristics in relation to patient age.

Lesion Morphology	>85	76–85	65–75	*p*-Value
**blisters**	91.67% (11)	76.19% (16)	86.36% (19)	0.4640
**papules**	16.67% (2)	4.76% (1)	23.81% (5)	0.2165
**erosion**	100.00% (11)	71.43% (15)	57.14% (12)	0.0381
**pruritus**	62.50% (5)	60.00% (12)	75.00% (15)	0.5806
**erythema**	90.91% (10)	95.24% (20)	85.00% (17)	0.5380

**Table 4:** Review of treatment methods in males and females.

Treatment	Males	Females	*p*-Value
**topical steroids**	50.00% (11)	48.48% (16)	0.8688
**systemic steroids**	40.91% (9)	48.48% (16)	0.7823
**methotrexate**	31.82% (7)	39.39% (13)	0.7748
**tetracycline + vitamin pp**	27.27% (6)	24.24% (8)	0.9496
**antibiotics other**	13.64% (3)	6.06% (2)	0.6321

**Table 5:** Review of treatment methods in relation to patient age.

Treatment	>85	76–85	65–75	*p*-Value
**topical steroids**	83.33% (10)	28.57% (6)	50.00% (11)	0.0102
**systemic steroids**	50.00% (6)	38.10% (8)	50.00% (11)	0.6900
**methotrexate**	33.33% (4)	33.33% (7)	40.91% (9)	0.8490
**tetracyclin +pp**	25.00% (3)	19.05% (4)	31.82% (7)	0.6297
**antibiotics other**	0.00% (0)	4.76% (1)	18.18% (4)	0.1440

## Data Availability

The data presented in this study are available on request from the corresponding author. The data are not publicly available due to privacy issues.

## References

[1] Miyamoto D., Santi C.G., Aoki V., Maruta C.W. (2019). Bullous pemphigoid. An. Bras. De Dermatol..

[2] Schmidt E., Zillikens D. (2013). Pemphigoid diseases. Lancet.

[3] Taquin H., Chiaverini C., Lacour J.P. (2016). Spectrum of Clinical Responses to therapies in infantile bullous pemphigoid. Pediatr. Dermatol..

[4] Lamb P.M., Abell E., Tharp M., Frye R., Deng J.-S. (2006). Prodromal bullous pemphigoid. Int. J. Dermatol..

[5] Lamberts A., Meijer J.M., Jonkman M.F. (2018). Nonbullous pemphigoid: A systematic review. J. Am. Acad. Dermatol..

[6] Cozzani E., Gasparini G., Burlando M., Drago F., Parodi A. (2015). Atypical presentations of bullous pemphigoid: Clinical and immunopathological aspects. Autoimmun. Rev..

[7] Meijer J.M., Diercks G.F.H., de Lang E.W.G., Pas H.H., Jonkman M.F. (2019). Assessment of diagnostic strategy for early recognition of bullous and nonbullous variants of pemphigoid. JAMA Dermatol..

[8] Zhang Y., Luo Y., Han Y., Tian R., Li W., Yao X. (2017). Non-bullous lesions as the first manifestation of bullous pemphigoid: A retrospective analysis of 181 cases. J. Dermatol..

[9] Ujiie H., Nishie W., Shimizu H. (2011). Pathogenesis of bullous pemphigoid. Dermatol. Clin..

[10] Kridin K., Ludwig R.J. (2018). The Growing Incidence of Bullous Pemphigoid: Overview and Potential Explanations. Front. Med..

[11] Milani-Nejad N., Zhang M., Kaffenberger J. (2017). The association between bullous pemphigoid and neurological disorders: A systematic review. Eur. J. Dermatol..

[12] Lloyd-Lavery A., Chi C.C., Wojnarowska F., Taghipour K. (2013). The associations between bullous pemphigoid and drug use: A UK case-control study. JAMA Dermatol..

[13] Wilczek A., Sticherling M. (2006). Concomitant psoriasis and bullous pemphigoid: Coincidence or pathogenic relationship?. Int. J. Dermatol..

[14] Sekiya A., Kodera M., Yamaoka T., Iwata Y., Usuda T., Ohzono A., Yasukochi A., Koga H., Ishii N., Hashimoto T. (2014). A case of lichen planus pemphigoides with autoantibodies to the NC16a and C-terminal domains of BP180 and to desmoglein-1. Br. J. Dermatol..

[15] Kalinska-Bienias A., Lukowska-Smorawska K., Jagielski P., Kowalewski C., Wozniak K. (2017). Mortality in bullous pemphigoid and prognostic factors in 1st and 3rd year of follow-up in specialized centre in Poland. Arch. Dermatol. Res..

[16] Bernard P., Antonicelli F. (2017). Bullous Pemphigoid: A Review of its Diagnosis, Associations and Treatment. Am. J. Clin. Dermatol..

[17] Marazza G., Pham H.C., Schärer L., Pedrazzetti P.P., Hunziker T., Trüeb R.M., Hohl D., Itin P., Lautenschlager S., Naldi L. (2009). Autoimmune bullous disease Swiss study group. Incidence of bullous pemphigoid and pemphigus in Switzerland: A 2-year prospective study. Br. J. Dermatol..

[18] Meijer J.M., Lamberts A., Luijendijk H.J., Diercks G.F.H., Pas H.H., Zuidema S.U., Jonkman M.F. (2019). Prevalence of Pemphigoid as a Potentially Unrecognized Cause of Pruritus in Nursing Home Residents. JAMA Dermatol..

[19] Murphy W.G. (2014). The sex difference in haemoglobin levels in adults—mechanisms, causes, and consequences. Blood Rev..

[20] Prati D., Taioli E., Zanella A., Della Torre E., Butelli S., Del Vecchio E., Vianello L., Zanuso F., Mozzi F., Milani S. (2002). Updated definitions of healthy ranges for serum alanine aminotransferase levels. Ann. Intern. Med..

[21] Bağcı I.S., Horváth O.N., Ruzicka T., Sárdy M. (2017). Bullous pemphigoid. Autoimmun. Rev..

[22] Zierk J., Krebs A., Rauh M., Metzler M., Löscher A., Strasser E., Krause S.W. (2020). Blood counts in adult and elderly individuals: Defining the norms over eight decades of life. Br. J. Haematol..

[23] Briand C., Gourier G., Poizeau F., Jelti L., Bachelerie M., Quéreux G., Jeudy G., Acquitter M., Ingen-Housz-Oro S., Caux F. (2020). Characteristics of Pruritus in Bullous Pemphigoid and Impact on Quality of Life: A Prospective Cohort Study. Acta Derm. Venereol..

[24] Kalinska-Bienias A., Kowalczyk E., Jagielski P., Lesniewska A., Komorowska A., Kowalewski C., Wozniak K. (2020). Clinical characteristics of pruritus in patients with bullous pemphigoid: A preliminary questionnaire-based study. Postępy Dermatol. I Alergol..

[25] Tominaga M., Ozawa S., Tengara S., Ogawa H., Takamori K. (2007). Intraepidermal nerve fibers increase in dry skin of acetone-treated mice. J. Dermatol. Sci..

[26] Swarna S.S., Aziz K., Zubair T., Qadir N., Khan M. (2019). Pruritus Associated with Chronic Kidney Disease: A Comprehensive Literature Review. Cureus.

[27] Sun C., Feng S. (2021). Recent developments in the pathogenesis of pruritus in bullous pemphigoid. Int. J. Dermatol..

[28] Fang H., Zhang Y., Li N., Wang G., Liu Z. (2018). The Autoimmune Skin Disease Bullous Pemphigoid: The Role of Mast Cells in Autoantibody-Induced Tissue Injury. Front. Immunol..

[29] Zebrowska A., Wagrowska-Danilewicz M., Danilewicz M., Stasikowska-Kanicka O., Kulczycka-Siennicka L., Wozniacka A., Waszczykowska E. (2014). Mediators of mast cells in bullous pemphigoid and dermatitis herpetiformis. Mediat. Inflamm..

[30] Hashimoto T., Kursewicz C.D., Fayne R.A., Nanda S., Shah S.M., Nattkemper L., Yokozeki H., Yosipovitch G. (2020). Pathophysiologic mechanisms of itch in bullous pemphigoid. J. Am. Acad. Dermatol..

[31] Rai P. (2023). Role of neutrophil-to-lymphocyte, neutrophil-to-eosinophil and platelet-to-lymphocyte ratios in the diagnosis of bullous pemphigoid and Pemphigus disease. Indian J. Pathol. Microbiol..

[32] Kremer N., Zeeli T., Sprecher E., Geller S. (2017). Failure of initial disease control in bullous pemphigoid: A retrospective study of hospitalized patients in a single tertiary center. Int. J. Dermatol..

[33] Kwan Z., Lai Y.N., Ch’ng C.C., Tan A.H., Tan L.L., Robinson S., Rokiah I. (2015). The association between bullous pemphigoid and neurological disorders in a selected Malaysian population. Med. J. Malays..

[34] Kalińska-Bienias A., Kowalczyk E., Jagielski P., Bienias P., Kowalewski C., Wozniak K. (2019). The association between neurological diseases, malignancies and cardiovascular comorbidities among patients with bullous pemphigoid: Case-control study in a specialized Polish center. Adv. Clin. Exp. Med..

[35] Yan T.M., Zuo Y.G. (2019). Coagulation Disorders in Bullous Pemphigoid and Its Mechanism. Acta Acad. Med. Sin..

[36] Bech R., Kibsgaard L., Vestergaard C. (2018). Comorbidities and Treatment Strategies in Bullous Pemphigoid: An Appraisal of the Existing Litterature. Front. Med..

[37] Geller S., Kremer N., Zeeli T., Sprecher E. (2018). Bullous pemphigoid and diabetes mellitus: Are we missing the larger picture?. J. Am. Acad. Dermatol..

[38] Chuang T.Y., Soltani K., Clayman J., Clayman J., Cook J. (1984). Increased frequency of diabetes mellitus in patients with bullous pemphigoid: A case-control study. J. Am. Acad. Dermatol..

[39] Tasanen K., Varpuluoma O., Nishie W. (2019). Dipeptidyl peptidase-4 inhibitor-associated bullous pemphigoid. Front. Immunol..

[40] Atzmony L., Mimouni I., Reiter O., Leshem Y.A., Taha O., Gdalevich M., Hodak E., Mimouni D. (2017). Association of bullous pemphigoid with malignancy: A systematic review and meta-analysis. J. Am. Acad. Dermatol..

[41] Lucariello R.J.E., Villablanca S., Mascaró J., Reichel M. (2018). Association between bullous pemphigoid and malignancy: A meta-analysis. Australas. J. Dermatol..

[42] Brick K.E., Weaver C.H., Savica R., Lohse C.M., Pittelkow M.R., Boeve B.F., Gibson L.E., Camilleri M., Wieland C.N. (2014). A population-based study of the association between bullous pemphigoid and neurologic disorders. J. Am. Acad. Dermatol..

[43] Pietkiewicz P., Gornowicz-Porowska J., Bowszyc-Dmochowska M., Bartkiewicz P., Dmochowski M. (2016). Bullous pemphigoid and neurodegenerative diseases: A study in a setting of a Central European university dermatology department. Aging Clin. Exp. Res..

[44] Tarazona M.J., Mota A.N., Gripp A.C., Unterstell N., Bressan A.L. (2015). Bullous pemphigoid and neurological disease: Statistics from a dermatology service. An. Bras. Dermatol..

[45] Jedlickova H., Hlubinka M., Pavlik T., Semradova V., Budinska E., Vlasin Z. (2010). Bullous pemphigoid and internal diseases: A case-control study. Eur. J. Dermatol..

[46] Langan S.M., Groves R.W., West J. (2011). The relationship between neurological disease and bullous pemphigoid: A population-based case-control study. J. Investig. Dermatol..

[47] Sadik C.D., Schmidt E. (2019). Resolution in bullous pemphigoid. Semin. Immunopathol..

[48] Taghipour K., Chi C.-C., Bhogal B., Groves R.W., Venning V., Wojnarowska F. (2014). Immunopathological characteristics of patients with bullous pemphigoid and neurological disease. J. Eur. Acad. Dermatol. Venereol..

[49] Seppänen A., Autio—Harmainen H., Alafuzoff I., Särkioja T., Veijola J., Hurskainen T., Bruckner-Tuderman L., Tasanen K., Majamaa K. (2006). Collagen XVII is expressed in human CNS neurons. Matrix Biol..

[50] Seppänen A., Suuronen T., Hofmann S.C., Majamaa K., Alafuzoff I. (2007). Distribution of collagen XVII in the human brain. Brain Res..

[51] Kokkonen N., Herukka S.K., Huilaja L., Kokki M., Koivisto A.M., Hartikainen P., Remes A.M., Tasanen K. (2017). Increased levels of the bullous pemphigoid BP180 autoantibody are associated with more severe dementia in Alzheimer’s disease. J. Investig. Dermatol..

[52] Künzli K., Favre B., Chofflon M., Borradori L. (2016). One gene but different proteins and diseases: The complexity of dystonin and bullous pemphigoid antigen 1. Exp. Dermatol..

[53] Seppänen A. (2013). Collagen XVII: A shared antigen in neurodermatological interactions?. Clin. Dev. Immunol..

[54] Ujiie H. (2023). What’s new in the pathogeneses and triggering factors of bullous pemphigoid. J. Dermatol..

[55] Di Zenzo G., Thoma-Uszynski S., Calabresi V., Fontao L., Hofmann S.C., Lacour J.-P., Fontao L., Hofmann S.C., Lacour J.P., Sera F. (2011). Demonstration of epitope-spreading phenomena in bullous pemphigoid: Results of a prospective multicenter study. J. Investig. Dermatol..

[56] Verheyden M.J., Bilgic A., Murrell D.F. (2020). A Systematic Review of Drug-Induced Pemphigoid. Acta Derm. Venereol..

[57] Benzaquen M., Borradori L., Berbis P., Cazzaniga S., Valero R., Richard M.A., Feldmeyer L. (2018). Dipeptidyl peptidase IV inhibitors, a risk factor for bullous pemphigoid: Retrospective multicenter case-control study from France and Switzerland. J. Am. Acad. Dermatol..

[58] Ricci M., Zauli S., Zelante A., Trevisani L., Virgili A., Bettoli V. (2014). Bullous pemphigoid occurring under anti-tumor necrosis factoralpha therapy. Int. J. Color. Dis..

[59] Dănescu S., Chiorean R., Macovei V., Sitaru C., Baican A. (2015). Role of physical factors in the pathogenesis of bullous pemphigoid: Case report series and a comprehensive review of the published work. J. Dermatol..

[60] Ceryn J., Skibińska M., Barasińska P., Noweta M., Narbutt J., Lesiak A. (2022). UVB-induced bullous pemphigoid in a patient with psoriasis. Adv. Dermatol. Allergol./Postępy Dermatol. I Alergol..

[61] Ghanaatpisheh A., Safari M., Haghshenas H., Motamed-Sanaye A., Atefi A.H., Kamangarpour K., Bagherzadeh M.A., Kamran-Jahromi A., Darayesh M., Kouhro N. (2024). New-onset or flare-up of bullous pemphigoid associated with COVID-19 vaccines: A systematic review of case report and case series studies. Front. Med..

[62] Borradori L., Van Beek N., Feliciani C., Tedbirt B., Antiga E., Tedbirt B., Antiga E., Bergman R., Böckle B.C., Caproni M. (2022). Updated S2 K guidelines for the management of bullous pemphigoid initiated by the European Academy of Dermatology and Venereology (EADV). J. Eur. Acad. Dermatol. Venereol..

